# Cerebral metabolism in a mouse model of Alzheimer’s disease characterized by two-photon fluorescence lifetime microscopy of intrinsic NADH

**DOI:** 10.1117/1.NPh.5.4.045008

**Published:** 2018-12-27

**Authors:** Carlos A. Gómez, Buyin Fu, Sava Sakadžić, Mohammad A. Yaseen

**Affiliations:** a Massachusetts General Hospital, Harvard Medical School, Athinoula A. Martinos Center for Biomedical Imaging, Department of Radiology, Charlestown, Massachusetts, United States

**Keywords:** Alzheimer’s disease, cerebral metabolism, fluorescence lifetime imaging, microscopy, phasors

## Abstract

Disruptions and alterations to cerebral energy metabolism play a vital role in the onset and progression of many neurodegenerative disorders and cerebral pathologies. In order to precisely understand the complex alterations underlying Alzheimer’s disease (AD) progression, *in vivo* imaging at the microscopic level is required in preclinical animal models. Utilizing two-photon fluorescence lifetime imaging microscopy and the phasor analysis method, we have observed AD-related variations of endogenous fluorescence of reduced nicotinamide adenine dinucleotide (NADH) *in vivo*. We collected NADH FLIM images from the cerebral cortices of both APPswe:PS1dE9 mice to model amyloid β plaque accumulation and corresponding age-matched wildtype controls. Distinct variations in NADH fluorescence lifetime between wildtype and AD mice, as well as variations related to proximity from amyloid plaques, are obvervable via the phasor method. The combination of NADH FLIM and phasor analysis allows for a minimally invasive, high-resolution technique to characterize the adverse effects of amyloid β accumulation on mitochondrial energy metabolism and could guide our understanding of preclinical AD pathology.

## 1 Introduction

Alzheimer’s disease (AD) is a devastating neurodegenerative disease with rapidly increasing incidence. Current estimates indicate that AD accounts for 60% to 80% of dementia cases,^1^ and projections estimate that it will affect as many as 74.7 million people worldwide by 2030, costing ∼$2 trillion for treatment and care.^2^ Presently, AD is diagnosed clinically when behavioral symptoms such as memory loss and deteriorating cognition manifest in patients. A “prodromal” phase of AD progression reportedly initiates as much as 20 years in advance of outwardly observable behavioral and cognitive deficiencies. Hallmark preclinical changes that begin during the prodromal phase include accumulation of soluble amyloid β (Aβ) oligomers, insoluble Aβ plaques, neurofibrillary tangles from misfolded tau protein, and activated microglia and astrocytes.^3^^,^^4^ These pathological features are associated with notable impairments to glucose metabolism, cerebral blood flow, and mitochondrial function. In addition, numerous Aβ-induced alterations to mitochondrial structure and function have been observed.^5^^–^^8^ The ability to monitor cerebral metabolism is vitally important to understanding AD’s trajectory and to develop robust biomarkers and therapeutic approaches. Preclinical AD-related changes that manifest at a cellular- and neurovascular-level requires minimally invasive, high-resolution techniques for detailed characterization.

For several decades, measurements of reduced nicotinamide adenine dinucleotide (NADH) have been utilized as a nondestructive tool to characterize mitochondrial respiratory chain activities. NADH is an electron carrier molecule residing in almost all eukaryotic cells both in cytosol and mitochondria, and it performs vital roles in both anaerobic glycolysis and aerobic oxidative metabolism.^9^ Extensive studies have utilized single photon, UV excitation of NADH fluorescence in isolated mitochondria or cell suspensions as a sensitive, quantitative measure of oxygen-dependent mitochondrial respiratory chain activity.^10^^–^^12^ Under these *in vitro* conditions, oxygen levels can be adjusted and metabolic substrates can be added such that the sample’s NADH can reversibly be adjusted between its fully oxidized and fully reduced states, therefore providing calibration for the minimal and maximal detectable NADH fluorescence signal. Confounds arising from toxicity prevent calibration and absolute quantitation in living organisms; however, evaluating relative changes of *in vivo* NADH fluorescence intensity still provides a useful indication of pathology-induced variations of mitochondrial function and intracellular oxygen requirements.^13^ The *in vivo* measurement techniques’ utility was greatly extended by coupling NADH fluorescence with 2-photon microscopy (2PM),^14^^,^^15^ a powerful tool for investigating multiple facets of brain function with high spatial and temporal resolution using deeper penetrating near-infrared excitation.^16^^,^^17^ While fluorescence intensity imaging of NADH offers a nondestructive, label-free, and simple method to evaluate mitochondrial function, fluorescence lifetime imaging microscopy (FLIM) shares these benefits and is also insensitive to fluorophore concentration or excitation and emission intensity. By measuring the time-resolved fluorescence decay at each pixel, FLIM provides a robust method to evaluate microenvironmental conditions around a fluorophore, including changes in temperature, pH, or in the case of NADH, alterations in enzymatic binding.^18^^,^^19^ FLIM-based monitoring of NADH offers considerable potential for assessing more subtle variations in mitochondrial energy metabolism with subcellular resolution, particularly when it is integrated with 2PM.^20^^–^^24^ In our previous investigations, we used an assortment of pharmacological inhibitors to validate the hypothesis that *in vivo* FLIM measurements can resolve disruptions to the TCA cycle, electron transport chain, and oxidative phosphorylation.^25^ Our earlier studies utilized traditional FLIM analysis methods based on nonlinear curve-fitting algorithms, which are computationally intensive and often exceedingly sensitive to potential inaccuracies. As an alternative to nonlinear curve-fitting methods, the polar plot (aka “phasor”) analysis method offers an elegant, Fourier-based alternative approach that requires less computation power and makes no assumptions about the mathematical models of the fluorescence lifetime decays.^26^^–^^29^ Variations in fluorescence lifetime can easily be visualized, indicated by differences in location on the two-dimensional (2-D) phasor plot. We previously demonstrated that phasor analysis of endogenous NADH fluorescence can robustly detect pharmacologically induced alterations in mitochondrial energy metabolism.^30^ Shifts in the location on the phasor plots indicated disruptions to the TCA/Krebs cycle, the electron transport chain, and oxidative phosphorylation, and we found that the phasor technique enables development of improved models for classifying metabolic perturbations.

In this report, we apply two-photon fluorescence lifetime imaging microscopy (2P-FLIM) of endogenous cortical NADH for minimally invasive characterization of AD-induced variations in cerebral metabolism in a mouse model of AD. We utilized phasor analysis to show that amyloid β plaques cause significant variations to fluorescence lifetime of endogenous NADH. In addition, we observed AD-related variations to spatial distribution and fluorescence lifetime of the aging pigment, lipofuscin. The results show promise to guide our understanding of the metabolic alterations associated with AD progression.

## 2 Materials and Methods

Our imaging experiments were performed under a protocol approved by the Institutional Animal Care and Use Committee at Massachusetts General Hospital. For our studies, we utilized female transgenic APPswe:PS1dE9 mice (N=7) and their age-matched wildtype controls (N=4) (8 to 13 months, 22 to 30 g). The APPswe:PS1de9 mouse model displays elevated soluble Aβ content and Aβ plaques by 4 to 6 months of age, with progressive Aβ accumulation thereafter.^31^ A 3-mm cranial window was surgically implanted into the skull and was held in place by acrylic. Mice were placed under isoflurane anesthesia and their physiological conditions (heart rate, temperature, and arterial blood gases) were monitored. Aβ plaques were fluorescently labeled by topical application of trypan blue. 2P-FLIM images were collected using a custom-made multimodal microscope which features a titanium:sapphire laser (Insight DeepSee, Spectra Physics, excitation wavelength tuned to $\lambda_{\mathrm{ex}}$: 740 nm, repetition frequency f=80  MHz), high-numerical aperture objective lens (Olympus XLumPlan Fluor, 20×, 1.00 NA, 2 mm working distance), galvanometer-based scanner mirrors (6215HB, Cambridge Technology, Inc.), and commercial electronics for time-correlated single-photon counting (SPC-150, DCC,-100, GVD-120, Becker & Hickl GmbH).^19^^,^^32^ For these experiments, commercial SPCM software from Becker & Hickl was used for the acquisition. Incident power varied but remained well below 50 mW for all measurements. 2P-FLIM measurements consisted of repetitive 256×256  pixel raster scans performed over 80 to 150  μm fields of view at frame intervals of up to ∼900  ms for 120 s. Each recorded measurement consisted of a 256×256×256 matrix of photon counts (x,y,t; Δt≈50  ps binning intervals) for NADH and trypan blue emission. A hybrid photomultiplier tube (PMT) with high detection efficiency and minimal afterpulsing (HPM-100-40 Becker & Hickl, GmbH) was used to collect NADH emission, and trypan blue fluorescence was detected using a PMT. Compared to NADH autofluorescence (emission wavelength $\lambda_{\mathrm{em}}∼450\;\mathrm{nm}$), the emission peak for trypan blue is redshifted by over 100 nm. We verified that trypan blue fluorescence is not seen by our NADH detection channel.

All FLIM data were analyzed using customized software developed in Matlab (Mathworks Inc., Natick, Massachusetts). By integrating the photon count matrices along the time axis, intensity images were generated and used for preprocessing. First, to isolate parenchymal NADH fluorescence, features such as blood vessels were masked out manually, as well as constituents displaying endogenous fluorescence such as lipofuscin granules. The boundaries for Aβ plaques were also delineated manually using the trypan blue images. Fourier-based phasor calculations were performed on the 2P-FLIM fluorescence decay I(t),^26^^,^^27^ in order to get the phasor coordinates g(ω) and s(ω); where angular frequency ω relates to laser repetition frequency f as 2*π*f:

$$g(\omega )=\frac{\int_{0}^{\infty }I(t)\cos(\omega t)dt}{\int_{0}^{\infty }I(t)dt}\;s(\omega )=\frac{\int_{0}^{\infty }I(t)\sin(\omega t)dt}{\int_{0}^{\infty }I(t)dt}.$$

The coordinates, g(ω) and s(ω), were then plotted on a 2-D phasor map. The phasor coordinates for monoexponential decays lie along the “phasor universal circle,” the semicircle defined by the equation ${\left(g-0.5\right)}^{2}+s^{2}=0.25$ (s≥0). Meanwhile fluorescence profiles that exhibit multiexponential decays will fall within the semicircle, at locations that are the vectorial sum of the fractionally weighted individual terms. These calculations were performed by our custom software along with groupwise analysis of multiple samples and comparison of different regions of interest (ROI) including concentric annular ROIs. The software also assessed separability of different phasor clusters using the Bhattacharyya distance, computed as

$$D_{B}=\frac{1}{8}{\left(\mu_{1}-\mu_{2}\right)}^{T}{\left(\frac{P_{1}+P_{2}}{2}\right)}^{-1}(\mu_{1}-\mu_{2})+\frac{1}{2}\;\ln(\frac{\left|\frac{P_{1}+P_{2}}{2}\right|}{\left|P_{1}||P_{2}\right|}),$$

where $\mu_{i}$ and $P_{i}$ correspond to the mean coordinates and covariance matrix for each phasor cluster.^33^^,^^34^ Like the Mahalanobis distance,^20^ the Battacharyya distance is utilized to assess the similarity between two statistical distributions and is useful for characterizing the separability of different classes of data in multidimensional space as well as feature extraction and classification.^35^ It differs from the Mahalanobis distance by allowing for distributions with unequal covariance. If two distributions are identical, $D_{B}$ is zero. A larger statistical distance implies greater separability between classes. We designated $D_{B}\geq 0.9$ as the threshold value for significant separability between phasor clusters, as this value has been used to develop classification models that perform with high accuracy (∼90%).^36^

## 3 Results and Discussion

**Fig. 1:**
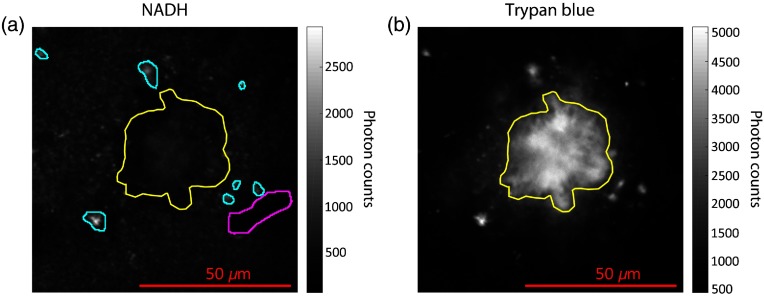
Intensity image of (a) endogenous NADH fluorescence and (b) corresponding fluorecence of topically applied trypan blue, collected *in vivo* from cortical tissue of an AD mouse model with different morphological features outlined: yellow, amyloid β plaque; teal, lipofuscin deposits; and magenta, cortical blood vessel. These features were masked for to isolate parenchymal NADH.

We sought to evaluate AD-related alterations to mitochondrial function by measuring NADH in the living cortex of AD mouse models. Similar to flavin adenine dinucleotide (FAD), NADH is endogenous and ubiquitous within most eukaryotic cells, and it participates in multiple steps of glucose breakdown and ATP synthesis. Monitoring the fluorescence of these electron carriers uniquely enables nondisruptive assessment of energy metabolism within living biological tissue.^9^ However, like most intrinsic fluorophores, both FAD and NADH have low two-photon action cross sections and are thus weakly excitable compared to exogenous dyes.^37^ Based on our previous efforts, additional experimental optimizations are necessary to overcome limitations before FAD fluorescence can be robustly measured in living brains, including weak excitation, absorption by hemoglobin, and suboptimal detection efficiency. Consequently, our characterizations of AD-related cerebral metabolic alterations focused primarily upon lifetime-based measurements of intrinsic NADH. First, this required isolation of parenchymal NADH signal from other morphological features such as blood vessels and endogenous constituents that also displayed fluorescence. Figures 1(a) and 1(b) show example fluorescence intensity images within and around an Aβ plaque in the living mouse cortex from detector channels used to measure NADH ($\lambda_{\mathrm{em}}=460\pm 30\;\mathrm{nm}$) and trypan blue ($\lambda_{\mathrm{em}}=595\pm 25\;\mathrm{nm}$), respectively. While topical application of trypan blue enabled easy and specific visualization of Aβ plaques and amyloid deposits along vascular segments, the plaques were also discernible by autofluorescence in the lower wavelength emission channel, even in the absence of trypan blue. Typically, the interior portions of plaques demonstrated autofluorescence with comparable intensity to the NADH from surrounding parenchymal tissue,^38^ but the plaques were enveloped by a dark band around the periphery where no signal was present. The aging pigment lipofuscin was also observed in both AD mice and their wildtype counterparts, appearing as bright, punctate crystals with broad emission detectible in both detector channels. In the AD mouse model, we consistently observed the high accumulation of lipofuscin crystals around the plaque boundary, as seen in Fig. 1(a). We isolated the NADH fluorescence from neuropil and cell bodies by manually masking out ROIs corresponding to blood vessels, autofluorescence from Aβ plaques, and lipofuscin granules.

**Fig. 2:**
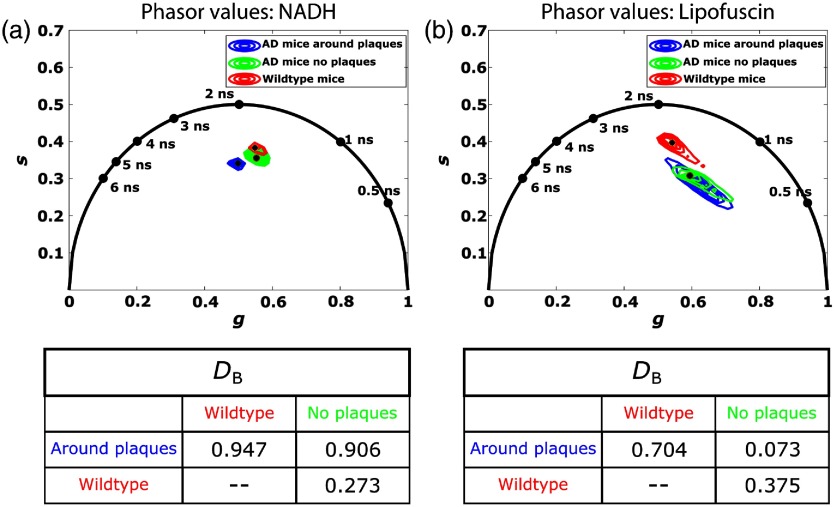
Phasor clusters for the parenchymal NADH and lipofuscin collected from AD mice and wildtype controls, and computed Bhattacharyya distances ($D_{B}$) of each cluster. The NADH phasor clusters of measurements taken near Aβ plaques appear well separated, suggesting that phasors can be reliably used to distinguish Aβ-related differences in cerebral metabolism. Lipofuscin measurements of wildtype mice appear distant from AD mouse measurements, although $D_{B}$ imply similarity.

After masking, phasor computations were performed on all measurements and grouped using our custom software. Figures 2(a) and 2(b) show phasor clusters corresponding to parenchymal NADH and lipofuscin, respectively, in wildtype and AD mouse models. The clusters are displayed as contour plots of the 2-D histograms (from 40% to 100% of the histograms peak centroid). We calculated phasor coordinates corresponding to cortical tissue measurements collected in wildtype mice (N=22 measurements from four mice), measurements from AD mice around an Aβ plaque (N=43 measurements from seven mice), and measurements from AD mice in fields of view where no plaques were present (N=6 measurements from four mice). NADH measurements from all mice localized in a similar region well inside the “phasor universal circle”^27^^,^^28^ as our previous measurements in Sprague Dawley rats,^30^ supporting repeated assertions that NADH exists as a population of different enzyme-bound formulations with different fluorescence lifetimes *in vivo* and *in vitro*. Conversely, lipofuscin phasor clusters were broad and dispersed over a range of short lifetimes. For both endogenous fluorophores, phasor clusters corresponding to measurements taken from regions near Aβ plaques appear visually separable from measurements taken far away from plaques or within wildtype mice with no plaques. For both NADH and lipofuscin measurements, we computed the Bhattacharyya distances ($D_{B}$) between each group to assess the separability between all clusters. As seen in Fig. 2, calculated $D_{B}$ distances for NADH around Aβ plaques exceeded our threshold criterion (>0.9) for statistical dissimilarity from the other groups, implying significant separability of NADH fluorescence lifetime in the presence of Aβ plaques. Lipofuscin measurements from wildtype mice appear well separated from AD mice on the phasor plots. However, computed $D_{B}$ distances fell below our designated threshold value and imply insignificant dissimilarity.

**Fig. 3:**
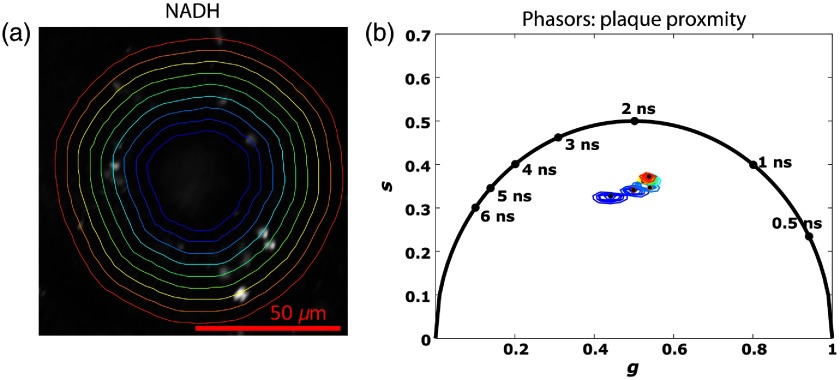
(a) Intensity image of an amyloid plaque and the surrounding cortical tissue with red delineated ROIs. All ROIs are 4-μm equidistant from each other. (b) Corresponding phasor clusters of the plaque (violet) and the eight cortical tissue ROIs. A distinct gradient is seen in the clusters’ centroids, varying with distance from the plaque.

To evaluate the spatial extent of metabolic variations around Aβ plaques, we partitioned NADH images into concentric annular ROIs at well-defined distances around the plaque border. Figure 3(a) shows an example partitioned NADH image with concentric band ROIs around a plaque. Each ROI corresponds to a 4-μm-thick region of tissue around the plaque boundary. Figure 3(b) shows the phasor clusters computed from inside an Aβ plaque and the surrounding annular regions from 43 plaque measurements. As seen by the upward shift in phasor centroids of more distal ROIs, a distinct gradient persists in the phasor clusters at distances up to 36  μm away from the plaque boundaries. Computed $D_{B}$ values for each ROI did not meet our criterion for significant cluster separability, which suggests that the influence of Aβ on mitochondrial function varies continuously rather than discretely as a function of proximity to plaques. Compared to more proximal tissue measurements, NADH measurements corresponding to tissue regions near the Aβ plaque show lower intensity with corresponding phasor clusters that are shifted downward and closer to the origin, similar to the effects induced by application of the respiration uncoupler carbonyl cyanide-4-(trifluoromethoxy)phenylhydrazone (FCCP).^25^^,^^30^ At increasing distances from plaques, the phasor clusters lie in areas corresponding to relatively shorter average lifetimes, similar to those of measurements taken in wildtype mice.

Although further validation is required, the present, phasor-based observations of endogenous NADH fluorescence from living cortices of AD mouse models strongly indicate a pronounced influence of Aβ plaques on mitochondrial energy metabolism. Specifically, the downward left shift in phasor location and lower fluorescence intensity of NADH near plaques suggests that Aβ interferes with oxidative phosphorylation. These observations agree well with *in vitro* observations of impaired respiration and mitochondrial toxicity induced by intracellular Aβ.^5^ The results are also consistent with previous reports, which utilized several exogenous contrast agents to demonstrate Aβ-induced impairments to mitochondrial prevalence and function. The dark bands surrounding plaques observed in our endogenous fluorescence images are in agreement with Xie et al.’s observations of mitochondrial loss near plaque borders, and our observed variations in NADH phasor clusters with plaque proximity support their observations of Aβ variations in mitochondrial membrane potential.^6^

Conversely, our observations of plaque-induced variations in lipofuscin fluorescence lifetime were unanticipated and raise intriguing questions regarding the nature and mechanisms of plaque-related toxicity. Often regarded as an aging-related pigment, lipofuscin granules are chemically and morphologically hetereogeneous masses primarily comprised of oxidatively modified protein and lipid residues that resist degradation by lysosomes. Lipofuscin is considered “biological garbage” that progressively accumulates within several tissues with age, but the rate of accumulation can be accelerated in the presence of higher oxygen or reduced by administration of antioxidants.^39^^,^^40^ Its presence is often overlooked or masked in brain imaging studies. Our observations of selective lipofuscin granule accumulation around plaque periphery and modest variation in fluorescence lifetime near plaques could reflect the vicious cycle of oxidative stress, mitochondrial impairments, Aβ accumulation, and plaque formation.^41^^,^^42^ Monitoring accumulation and fluorescence lifetime of lipofuscin could therefore be useful for assessing plaque formation and oxidative stress or toxicity associated with progression accumulation in the preclinical stages of AD.

Our study shows that phasor-based *in vivo* measurements of endogenous fluorescence are useful for characterizing distinct variations in mitochondrial metabolism and lipofuscin accumulation in the presence of Aβ plaques. The findings agree well with previous reports of metabolic alterations and present opportunities to noninvasively assess Aβ-related variations in oxidative stress and plaque formation. Metabolic function is distinctly affected in regions surrounding plaques, but it is unclear whether variations in mitochondrial function and oxidative stress are entirely attributable to amyloid β. Plaques are comprised primarily of improperly cleaved amyloid β protein, but they also contain a variety of other materials, including proteoglycans, metal ions, and inflammatory molecules such as cytokines.^43^ While soluble Aβ oligomers demonstrate notable neurotoxicity, our observations motivate further investigations to explore whether and how the additional plaque constituents contribute to metabolic impairments, oxidative stress, or autofluorescence by plaques.
